# Spousal emotional support and relationship quality buffers pupillary response to horror movies

**DOI:** 10.1371/journal.pone.0256823

**Published:** 2021-09-15

**Authors:** Tyler C. Graff, Joseph R. Fitzgerald, Steven G. Luke, Wendy C. Birmingham

**Affiliations:** 1 Department of Psychology, Brigham Young University, Provo, Utah, United States of America; 2 Department of Social Sciences, Wartburg College, Waverly, Iowa, United States of America; 3 School of Public Affairs, Arizona State University, Tempe, Arizona, United States of America; University of Milan, ITALY

## Abstract

Being satisfied in marriage provides protective stress buffering benefits to various health complications but the causal mechanisms and speed at which this is accomplished is less well understood. Much of the research on health and marriage has conceptualized marital quality in a unidimensional way, with high levels of either positivity or negativity. This conceptualization may not fully capture the nuanced benefits of marital relationships. Pupillometry is an innovative method which captures the effects of marital stress buffering on the body’s autonomic nervous system in real time; pupil dilation occurs within 200ms to stress exposure. Additionally, this method records hundreds of readings per second, providing precision and sensitivity. This preregistered experiment aimed to conceptually replicate previous pupillometry stress buffering results and extend the previous findings by including a generalizable, real-life stressor—viewing a horror movie—and multidimensional relationship quality effects. Eighty-three couples (166 participants) were quasi-grouped, based on a self-reported multidimensional relationship quality scale, to either supportive or ambivalent marital relationship conditions. They were then randomly assigned to either a spousal support (i.e., handholding) or non-support (spousal absence) condition and watched clips from both horror and nature movies while pupil dilation was measured. Tonic pupillary response results revealed that the horror video clips elicited a stress response and there were significant differences between the support and non-support conditions, as well as marital relationship quality conditions. These results frame the precision, speed, and sensitivity of pupillometry as a potentially fruitful method to investigate the causal mechanisms linking stress buffering and supportive marital relationships.

## Marriage and stress buffering

Epidemiological research has overwhelmingly demonstrated that supportive social relationships can protect individuals from various causes of morbidity and mortality [1–4]. The causal mechanisms and speed at which this is accomplished is less well understood. One way researchers have operationalized supportive social relationships is through couples’ sharing of emotional support which involves expressions of comfort and caring [5, 6]. Conveyed emotional support is a situational factor in response to trying, difficult, or stressful circumstances [6]. Providing emotional support can be seen as soothing, comforting, consoling, and a source of help during life’s challenges, communicating warmth, caring, and acceptance. Burleson [7] noted that emotional support can include non-verbal communicative behaviors (e.g., affectionate touch, hugs, handholding) that are enacted with the intent to help another cope. Emotional support conveyed through handholding elicits clear, non-verbal communication of comfort and security [5, 7]. The stress buffering model of social relationships posits that social support is primarily beneficial during periods of stress [3, 8–11]. Research has shown that during a stressor, simply imagined emotional support in the form of affectionate touch has stress buffering benefits [12] and physical handholding reduces anxiety associated with stressful events [13–15]. Research Question: Can the benefits associated with handholding be observed through a quick, objective, and physiological method?

One of the most important social connections for an adult is the relationship with a spouse, and spouses are often identified as the most important provider of emotional support [16, 17]. Emotional support from a spouse is beneficial in terms of relationship processes: provision of emotional support increases feelings of intimacy and promotes higher quality interpersonal interactions [18–20]. Provision of emotional support is also physiologically beneficial. Holt-Lunstad, Birmingham and Light [21] found emotional support provided through a warm touch intervention was associated with enhanced salivary oxytocin and reduced alpha amylase in both husbands and wives, and with lower systolic blood pressure for husbands. Coan et al. [14] found that emotional support in the form of handholding decreased neural responses to threat. This dampening effect was magnified if the person was holding the hand of their spouse. Importantly, the effect was even more powerful if the couple had a good marital relationship.

Years of research have definitively shown that on average married people have better psychological and physiological health than unmarried people across a variety of both chronic and acute health threats such as cancer, heart attacks, and surgery [22–24] and have lower rates of morbidity and mortality [25–28], However, quality matters. High marital quality buffers the harmful effects of lower socioeconomic status on blood pressure [29], while marital discord negatively impacts sleep quality and quantity, elevates risk for depression, increases the risk for developing metabolic syndrome, and increases risk of diabetes in women [30–33]. Indeed, Holt-Lunstad, Birmingham and Jones [34] found that marriage must be high quality, or one is better off single. Marital quality however is often seen on a unidimensional scale being either supportive (high support, low strain) or aggravating (low support, high strain), yet marriages can contain both high elements of support and high elements of strain concurrently (ambivalence). In a study evaluating 183 couples Reblin, Vaughn, Birmingham et al. [35] showed that the inclusion of multiple dimensions of relationship quality improved prediction of marital functioning. Recent research has examined ambivalence in marriage and found those who report spousal ambivalent behavior have worse psychological and physiological health, and worse relationship processes than marriages with supportive behaviors [29, 36–39]. Research Question: Will married couples’ relationship quality immediately influence a person’s physiological response?

### Stress and fear

In a biopsychological sense, stress can refer to anything, both physical and psychological, that puts a strain on an organism’s adaptive capabilities [40]. While the occasional acute stressor may not have any major negative impacts on overall functioning, chronic stress (stress lasting for an extended period of time) or incessant acute stressors can, over time, lead to diverse, negative health outcomes [41, 42]. These can range from increased susceptibility to the common cold [43] to increased healing time after surgery [44] to reduced receptibility of vaccines and greater vulnerability to disease [45]. Interestingly, stress and fear result in near identical physiological responses such as increased cardiovascular activity and pupil dilation [46–48] as well as residual feelings of anxiety [49, 50]. This is due to our bodies having a limited way to activate the nervous system in response to threat [40].

Humans have the unique ability to activate the stress or fear response to threats that are not imminent and even sometimes not real at all [51]. Horror films exploit this to stimulate very real fear and stress responses in movie-goers even when no real threat is present, eliciting physiological reactions just as robust as if the movie-goers were really threatened by whatever is on the screen [52–54]. Importantly, receiving emotional support can moderate the effects of stress or fear response via stress buffering [9, 10, 55, 56].

An important concept in our study is the conceptual definition of our stress task. Typical stressors can include active tasks where the participant does something or passive tasks where the participant does not perform in some way. Common active stressors are mental arithmetic, public speech, and the Stroop test [57] and common passive stressors include cold pressors or watching horror movies [52, 58]. Importantly, research has shown that active and passive stressors have differential effects on stress responses with active stressors evoking more salient cortisol, cardiovascular, and immune responses compared to passive stressors [57, 59, 60]. Although active stressors can evoke more reactivity, passive stressors also evoke physiological reactivity [46, 61]. For example, Hayashi et al. [52] used horror movie clips as a passive stressor and found that fear-induced stress can evoke a cardiovascular response. Passive stressors such as horror movies provide an ecologically valuable way to investigate stress reactivity. In our previous study [62], we demonstrated pupillary stress-buffering effects from an active task as we tracked participants’ pupil dilation while they actively completed a Stroop test either alone or with their spouse holding their hand. Results from our previous study indicated that participants’ pupils dilate less in response to an active stressor if their spouse concurrently holds their hand. In the present study, we used horror movie clips as our passive stressor to extend our previous findings. Importantly, blood pressure and heart rate changes usually occur within seconds of the onset of a stressor which give a more immediate effect of stressors both active and passive. In this way, cardiovascular reactivity served as an important supplementary measure for our primary focus of the study—pupillary response. Research Question: Will spousal handholding moderate the physiological effects of watching a horror movie?

### Autonomic nervous system: Pupillometry

When individuals become stressed, their pupils dilate [62, 63]. Pupillary fluctuations to cognitive stimuli are controlled by the autonomic nervous system. This includes reacting to stressors and enhancing the fight-or-flight response by the sympathetic nervous system, which can be indexed by pupil dilations [64]. The autonomic nervous system also includes relaxation to help the body return to regular functioning including homeostasis by the parasympathetic nervous system following a stressor as can be seen in pupil constriction. These pupillary changes to stimuli are controlled principally by the locus coeruleus (LC) [65]. The LC has been shown to have a significant role in autonomic activity; increases in LC activity are associated with more sympathetic arousal, whereas decreases in LC activity correlate with activation of the parasympathetic system [66]. The LC responds to stressors by secreting norepinephrine through the hypothalamic–pituitary–adrenal (HPA) axis. As the LC has a primary influence on pupil dilation, the measurement of pupil size can capture the immediate, real-time stress response.

In response to psychological stimuli, pupil dilations, typically smaller than 0.5 millimeters [67] also occur. From the onset of a stressor the pupils dilate within 200ms. After the event, the pupil size returns to baseline within about 2000ms [68]. Ren, Barreto, Huang et al. [69] showed that pupillometry is a robust and efficient method for detecting stress and that instrument reliability is comparable with traditional methods (e.g., skin conductance). Indeed, pupillometry has been a validated form of measuring stress/fear [70] and ANS activity [71–75]. Thus, stress and fear in this study are operationally defined as the acute physiological reaction of both tonic and phasic pupil dilation.

## Present study

The present experiment was designed to address if emotional support in the form of handholding from one’s spouse during a stressor affects the acute stress response as measured by pupil dilation. The study further explores whether the quality of the marital relationship significantly moderates the stress-reducing effect of spousal support. The present study conceptually replicates our previous pupillometry stress buffering results of spousal emotional support [62]. The initial study was the first to investigate the stress buffering benefits of spousal emotional support on pupil dilation by performing typical lab tasks associated with active stressors (i.e., the Stroop Task). In the present study, we expanded to use a generalizable, real-life, passive stressor (horror movies). We also extended these results using multidimensional relationship quality effects as we quasi-assigned participants to either the “supportive” or “ambivalent” relationships quality condition depending on their responses to prescreening using the Social Relationship Index (SRI).

### Hypotheses

In accordance with the research questions described above we hypothesize that:

1. When exposed to the experimental stressor (i.e., horror video clips), participants will demonstrate increased pupil dilation compared to control (i.e., neutral video clips).
2. Individuals who receive emotional support (Support Condition), in the form of handholding, from their spouse during the stress task will show a weaker pupillary stress response compared to individuals that do not receive emotional support from their spouse (Non-Support Condition). In other words, Stressor (Neutral vs. Horror video clips) will interact with Spouse Condition (Non-Support vs. Support).
3. Individuals in supportive marital relationships will benefit more from spousal emotional support than those in ambivalent relationships (three-way interaction between Relationship Quality, Video Condition, and Spouse Condition).

## Method

### Preregistration

We pre-registered the full study including planned analyses on the Open Science Framework. This was done on November 14, 2018, prior to any data collection.

### Participants

We used purposive sampling methods to recruit participants to our study; digital flyers were posted to social media sites such as Facebook, Twitter, and Instagram. Physical flyers were posted around the University’s campus and the surrounding county communities including public areas such as libraries. Potential participants were sent a screening survey which we used for quasi-assignment and to determine eligibility. Potential participants reported their marital relationship quality by completing the SRI during the screening process. Using an aggregate score, couples were then classified as having either a supportive or ambivalent relationship (more information on the SRI can be found in Measures below). Previous work in our lab has shown that relationship quality in marriage stabilizes later in marriage with more consistent reports of ambivalent relationships [39]. Therefore, to be eligible for this study, participants had to be married at least two years and be at least 21 years of age. Additional restrictions for participation included normal or corrected-to-normal vision, normal color vision, and no existing eye conditions to control for potential pupillary confounds. Data collection began December 2018 and our preregistration specified 200 total participants (100 couples) however, due to Covid-19 concerns we stopped data collection February 2020, slightly before obtaining that target. Ultimately, 166 participants (83 couples) were recruited from a university in the mid-western United States and the surrounding area. Participating couples had been married for an average of 10 years (*SD =* 7.33, *Range* = 3–29) and the average participant age was 33.29 (*SD* = 8.97, *Range* = 21–61). Demographic information can be found in Table 1. In one couple, the husband participated but the wife failed to complete the entirety of the tasks as she indicated not feeling well and withdrew from the study, and her pupillary data were excluded. This study was approved by the university’s Institutional Review Board (IRB approval number X16422).

**Table 1:** Demographics.

	*Mean*	*SD*	Range	N	%
Age in years	33.29	8.97	21–61	166	100
Marriage length (years)	10.45	7.33	3–29	166	100
Ethnicity:					
White				142	85.54
Hispanic/Asian/Other				24	14.46
Education status:					
Partial/graduated college				109	65.66
Partial/graduated graduate school				57	34.34
Annual income:					
< 50,000				77	46.39
> 50,000				77	46.39
Prefer not to answer				12	7.23
Self-reported health:					
Bad/Poor/Fair				56	33.73
Good/Excellent				110	66.26

*Note*. SD = Standard Deviation.

### Procedures

This study protocol followed the procedures as outlined in Graff et al. [62]. Eligible couples were randomized at a 1:1 ratio to either the support or non-support condition. Upon written consent, participants completed pre-assessment surveys (see Measures, below, for detail on the surveys). In the support condition, the spouse of the participant was consented separately for their role as support giver. Additionally, couples were randomly assigned for either the wife to complete the participant role first with the husband completing the support providing role, or vice versa. After the participant completed the pre-assessment surveys, both spouses were then brought back together, and both were then fitted with blood pressure cuffs. One participant refused to wear the blood pressure cuff as per allowances in the consent form, so no blood pressure measures were gathered for this participant (however, the participant completed all other experimental tasks normally and her data is included in the primary pupil analyses). Three baseline readings were obtained, each one minute apart. After the baseline readings, the participant was positioned in front of the eye tracker and their support giving spouse was positioned across from them behind the eye tracker, so the supporting spouse was unable to see the screen (see S1 Fig).

**Fig 1:**
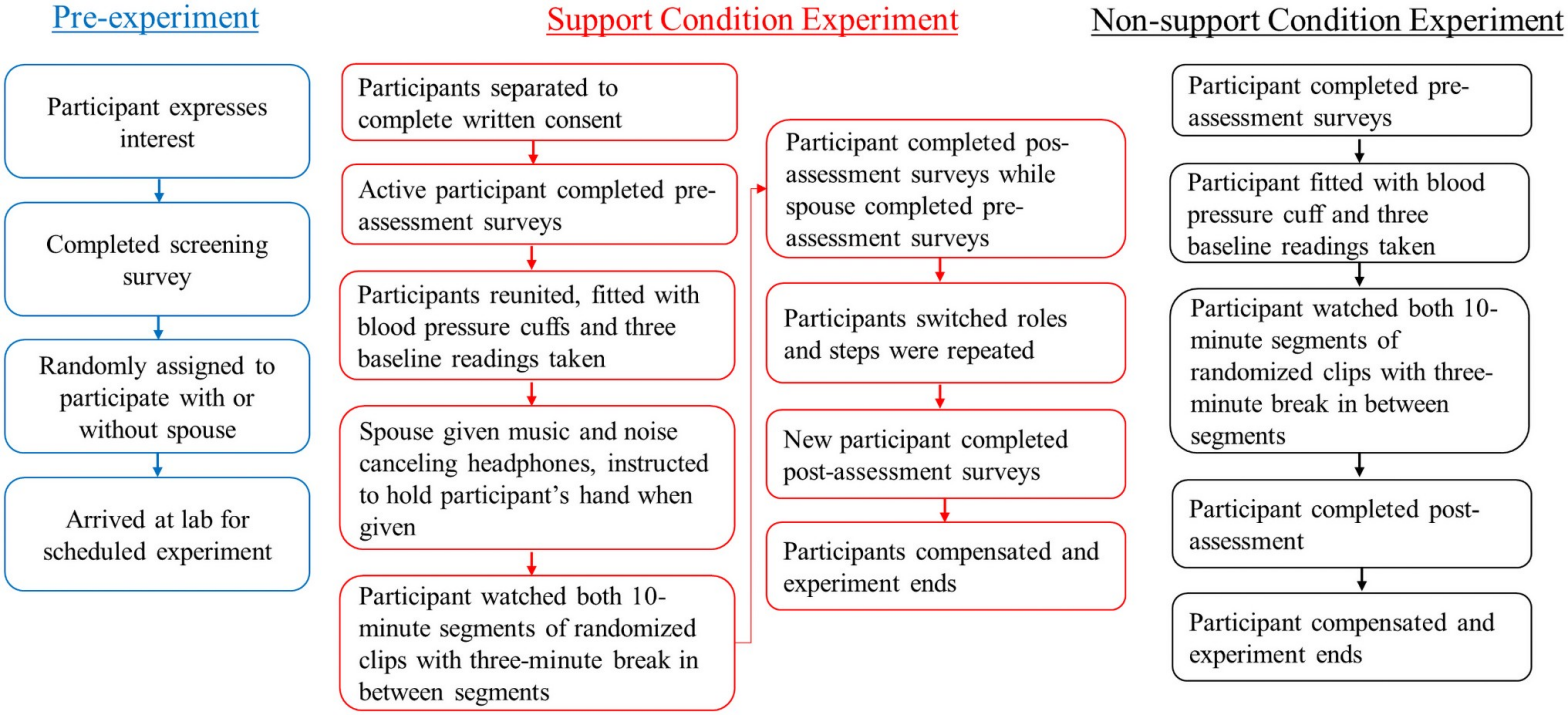
Experiment procedural flow.

Both the participant and the support giver were provided with noise cancelling headphones. The support giver selected a station on Pandora Music which they listened to throughout the procedure. The support giver was instructed not to distract the participant, including making faces, frequent fidgeting, teasing, and falling asleep. They were instructed to act as if they were providing support to their spouse. Finally, the participant and their support giving spouse held hands for the entirety of the task. To ensure that the support giver did not distract the participant and thereby confound the results, two research assistants ran each experiment, with one tasked explicitly to observe that the support giver was not being distracting and their data would not be included in the final analysis. All participants adhered to the experiment’s protocol (see Fig 1), and we did not have to remove any data from the final analysis due to these reasons.

The participant watched a set of video clips in two 10-minute segments with a brief (three minute) break between the two segments. To prevent order effects, each 10-minute segment was randomized for each couple to appear first or second. Before each segment, the eye tracker performed a 9-point calibration to the gaze of each participant. Participants looked at a small white dot on a gray background that moved across the screen to nine separate points all around the screen. This increases validity of obtained data and aids the eye tracker to recognize the unique gaze of each participant. These eye tracker calibrations took about 30 seconds. Blood pressure was taken during the last clip of each segment, for both the participant and the support giver. Importantly, pupil data were not recorded during these blood pressure readings to protect against pupillary confounds arising from the inflation of the cuff on the arm. After the first participant of the couple had finished the video procedure, they then completed a post-experiment survey, and the participant and the support giver switched places with the prior support giver becoming the new participant and the prior participant becoming the new support giver. They were each consented for their new roles and the video procedures were then completed by the new participant. The new support giver was once more explicitly told not to be distracting and was positioned so as not to be able to see the screen so they could not anticipate upcoming video segments and give more support to their spouse. In the non-support condition, spouses came in separately from each other (within 72 hours) and completed the tasks alone. Participants were asked not to discuss the study with their spouse until both had completed the experiment.

The video clips were taken from three different films; *I Know What You Did Last Summer* [76], *I Still Know What You Did Last Summer* [77], and *Alaska’s Wild Denali* [78]. These films were previously validated to elicit a certain response from viewers; the first two eliciting fear, and the last a neutral response [46, 61]. The selected clips were chosen from the longer sequences in Kreibig et al. [46] study, and each selected clip ranged in length from 31 seconds to 78 seconds. The scenes from the horror films all included at least one “frightening jump scare” designed to make the participant react, while the clips from neutral film produced no such response. In total, 22 clips were used, 11 each from the fear-inducing and neutral films (specific timestamps can be found in S1 File). The neutral clips were cut to be the same length as the horror clips so that a timestamp could be used to compare the pupil response between the fear-inducing clips and the neutral clips. The clips were randomly ordered and then divided into two groups to be shown as two segments. A few minor changes were made to the randomization to ensure that both segments were of the same approximate length. Between each clip, a large white fixation cross (+) was portrayed on the screen with a neutral gray background for one second. Participants looked at the center of the cross to get a stable tonic pupil size between each of the video clips. Additionally, because the pupillary light reflex is the main potential confound in cognitive pupillometry [79–81], the luminance of every aspect of our experimental conditions were controlled for (e.g., laboratory, stimuli, inter-stimuli luminance).

### Measures

A demographic questionnaire assessed basic variables including age, income, education level, and marital length.

#### Pupillometry tasks

Pupil dilation and constriction were recorded using the Tobii TX1200 eye-tracking system. A low intensity infrared light reflects off the participant’s retina, and infrared cameras then identify pupil size. Data and stimuli were recorded and stored using the Tobii Pro Lab software, which is designed to integrate with the TX1200. Eye position and pupil size were recorded at 1200Hz. The audio was controlled so that the decibel level did not exceed 77.3 decibels with an average decibel level of 66.6 decibels. This is well within safe volume for sound levels and helped prevent pupil dilation related to extraneously loud noises.

#### Social Relationship Index (SRI)

The SRI [82] is a multidimensional, self-reported scale used to measure both relationship positivity and negativity simultaneously. Respondents rated how positive and upsetting their spouse is as separate entities from each other. They rated on a 1 (not at all) to 6 (extremely) scale how positive and how upsetting their spouse is during daily interactions, during support seeking, and when the participant is in a happy mood. These responses are then aggregated to determine relationship quality types. Supportive is calculated with a medium to high positivity (2–6 on the positivity rating) and low upsetting (1 on upsetting rating). Ambivalent is calculated with a medium to high positivity as well as a concurrent medium to high upsetting (2–6 on upsetting rating) aggregate score. Previous work has shown high test-retest reliability after a two-week interval, with *r* = .81 (*p* < .001) for positivity and *r* = .83 (*p* < .001) for negativity [83] and Cronbach’s alpha from its validation study of.69 and.80 for positivity and negativity [82]. Indeed, the SRI evidenced acceptable internal consistency for positivity (Cronbach’s α = .72) and good internal consistency for upsetting (Cronbach’s α = .83) in the present study. Of the 166 participants in the study, 93 participants were classified as ambivalent and 73 were classified as supportive.

#### Spielberger State-Trait Anxiety Inventory (STAI)

We used a modified version of this inventory that included only 13 state anxiety questions. This measure has been used in previous fear inducement studies as measured by pupil dilation [84] and the STAI evidenced acceptable internal consistency (Cronbach’s α >.78) in its validation study (Marteau & Bekker, 1992). The measure was administered to participants both before and after viewing the videos. Participants rated their feelings on 13 questions with a 1 to 4 scale ranging from “not at all” to “very much” [85]. The STAI instrument evidenced good internal consistency (Cronbach’s α = .89).

#### Single item questions

These questions were derived from the work of Tomaka, Blascovich, Kibler et al. [86]. Both before and after watching the videos, participants responded to five questions about their feeling regarding the experiment on a 1 (not at all) to 6 (very much) scale. We asked participants how threatening, frightening, shocking, and stressful they expected the experiment to be and their same feelings after completion, as well their ability to cope with the experiment. In the post-experiment measures, participants also reported their frequency of watching horror movies and whether they had seen the movies before to control for variance in past exposure to stimuli. Additionally, we asked participants in the support condition if they found the handholding of their spouse while completing the tasks supportive.

### Statistical analysis plan

Following our preregistered plan (details can be found here: https://osf.io/2n6xm), we analyzed the pupillary data in two ways, tonic and phasic, using linear mixed-effects models [87] in R. *P*-values were obtained using the lmerTest package [88] by applying the Satterthwaite approximation for degrees of freedom [89]. All the statistical models included random intercepts for each video clip, as well as nested, random by-participant and by-couple intercepts with random slopes for all within-participant variables (Relationship Quality, Spouse Condition, Time, Horror or Neutral Clip), except when these random slopes prevented model convergence. We determined our significance levels by field-standard p-values (*p* < .05) and confidence intervals (95%).

Secondary analyses were performed as a physiological manipulation check for the pupillometry measure and included baseline and mid-task blood pressure. Additionally, subjective survey measures were used as another converging method as to whether participants perceived the study task as stressful/frightening. Pre and post self-report measures included the Spielberger state-trait anxiety scale [85] and the single item pre and post questions [86] and descriptive post experiment questions including: “did you find your spouse’s handholding supportive”, and “how often do you watch horror films”.

Two-way ANOVA’s with one repeated measure (Time) was conducted to compare pre- and post-cardiovascular measures and subjective task surveys. We also examined correlations between cardiovascular and self-report data.

## Results

### Secondary analyses

Descriptive single-item questions revealed that nearly all participants had not seen any of the video clips (87.2.8%, *n* = 116) and that only about a quarter of participants regularly watched horror movies (27.3%, *n* = 45). Of those in the support condition, nearly two thirds confirmed that the handholding of their spouse was supportive (69.1%. *n* = 47). Participants reported anticipating the movies to be only somewhat scary (*M* = 2.96, *SD* = 1.32) as well as similar ratings for other single item self-report questions (full self-report responses are included in Table 2).

**Table 2:** Mean self-report responses.

	Pre-Experiment		Post-Experiment	
	*M*	*SD*	*M*	*SD*
Perceived Threat	1.95	1.11	1.68	1.14
Perceived Ability to Cope	5.48	.95	5.50	1.12
Perceived Frightening	2.96	1.32	2.24	1.38
Perceived Shocking	2.77	1.30	2.37	1.39
Perceived Stressful	2.39	1.16	2.05	1.31
Spielberger State-Trait Anxiety Inventory	19.09	4.33	21.45	6.71

*Note*. M = Mean; SD = Standard Deviation. Perceived question scales range from 1 = not at all to 6 = very much. Spielberger state-trait anxiety scale ranges from 13–52, higher scores indicating higher anxiety.

**Fig 2:**
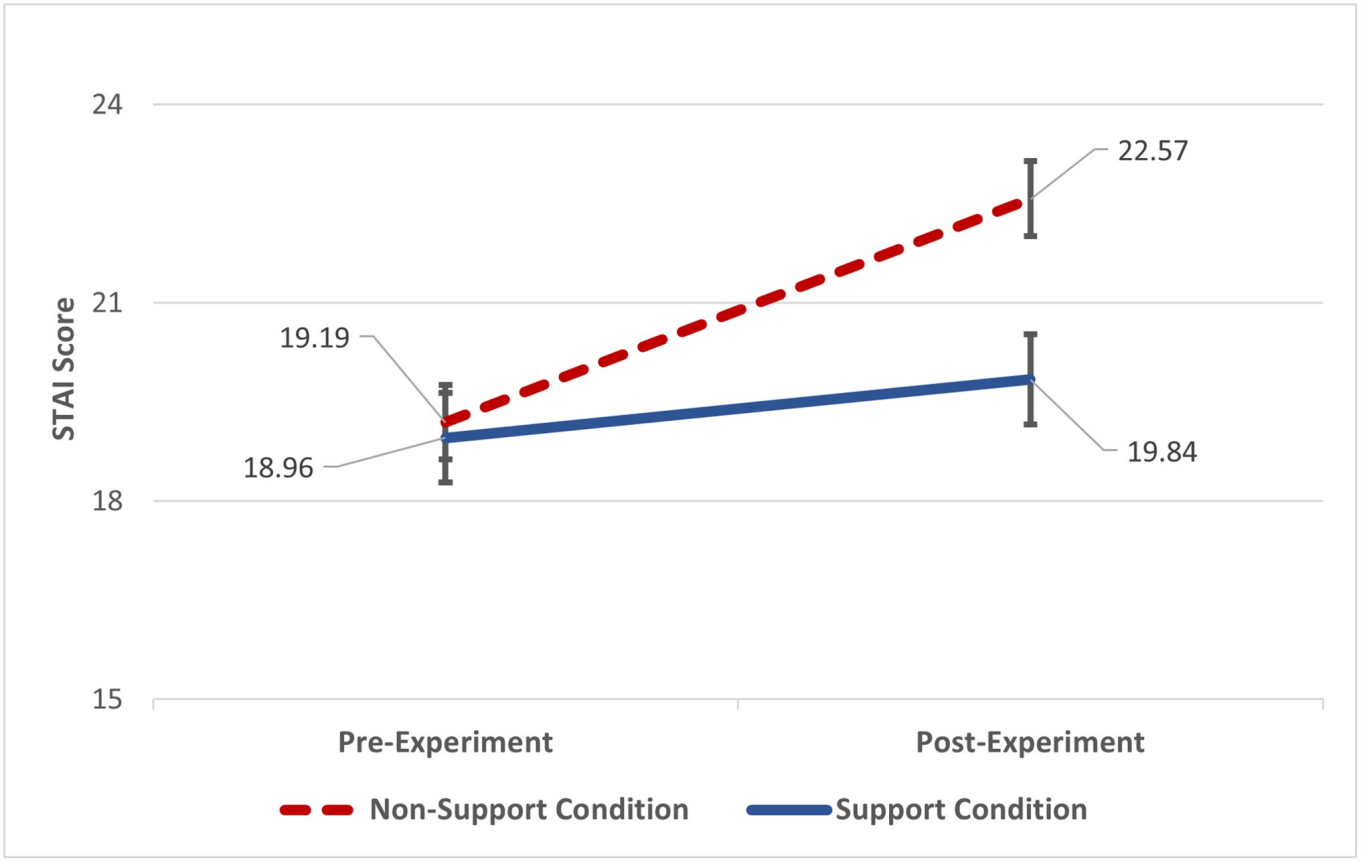
Interaction of Spielberger State-Trait Anxiety Inventory (STAI) and Spouse Condition. *Note*. Graph shows the interaction of participants’ self-reported pre anxiety and post anxiety scores and Spouse Condition. There were no differences in pre anxiety scores between the two conditions, but those in the non-support condition indicated higher feelings of anxiety post experiment compared to those who had their spouse hold their hand during the experiment. STAI scale ranges from 13–52, higher scores indicating higher anxiety.

Two-way ANOVA with one repeated measure (Time) were conducted to compare pre/post self-report measures and baseline/task systolic blood pressure (SBP), diastolic blood pressure (DBP), and pulse rate (PR). From the STAI, we found that participants’ self-reported pre anxiety score differed from their post anxiety score but only for the non-support condition (*F*(1, 163) = 7.46, *p* < .007, η^2^ = .044) indicating a calming effect of having one’s spouse hold their hand during the horror movies experiment (See Fig 2). We examined cardiovascular reactivity differences between the support and non-support conditions during the task as well as from baseline to task using repeated measures ANOVA. SBP results indicated no differences from baseline to task SBP (*F*(2, 322) = .72, *p* < .485) nor between support conditions (*F*(2, 322) = .33, *p* < .722). DBP and PR yielded similar outcomes. For the support condition, paired t-tests indicated significant differences in participant task SBP (*M* = 126.87, *SD* = 16.12) and support giver task SBP (*M* = 124.36, *SD* = 15.65; *t*(65) = 2.01, *p* < .048) and participant task DBP (*M* = 78.03, *SD* = 11.43) and support giver task DBP (*M* = 75.78, *SD* = 10.50; *t*(66) = 2.24, *p* < .028). This indicates that viewing the horror movies induced higher cardiovascular reactivity than simply providing support to one’s partner during the experiment. There were no differences between participant task and support giver task PR. Additionally, as part of our pre-registered secondary analysis plan, we examined associations between cardiovascular, self-report, and mean pupillary data. These correlations did not reveal any meaningful associations (see Table 3).

**Table 3:** Blood pressure, self-report, and pupil diameter correlations.

Variable	1	2	3	4	5	6	7	8
1. Sbp Experiment	-							
2. Dbp Experiment	0.75*	-						
3. Pr Experiment	0.09	0.11	-					
4. Self-Report Combined Items Pre-Experiment	0.11	0.09	0.16*	-				
5. Self-Report Combined Items Post-Experiment	-0.01	-0.01	0.04	0.54*	-			
6. STAI Pre-Experiment	0.13	0.17*	0.09	0.63*	0.36*	-		
7. STAI Post-Experiment	-0.01	0.04	0.06	0.47*	0.70*	0.49*	-	
8. Mean Pupil Change	-0.09	-0.01	0.09	-0.06	-0.11	-0.54	-0.18*	-

*Note*. Sbp = systolic blood pressure; Dbp = diastolic blood pressure; Pr = pulse rate; Self-Report combined items include threat, frightening, shocking, stress, and reverse-coded coping questions; STAI = Spielberger state-trait anxiety inventory; Mean Pupil Change = average pupil difference between neutral and horror videos.

* *p* < .05.

### Pupil analyses

Pupil dilation was recorded at 1200 Hz providing over 14 trillion total data points. We first averaged each participant’s data into 100ms bins. This allowed us to model both tonic and phasic pupil dilations and assess each of our *a priori* hypotheses.

#### Tonic analysis

**Fig 3:**
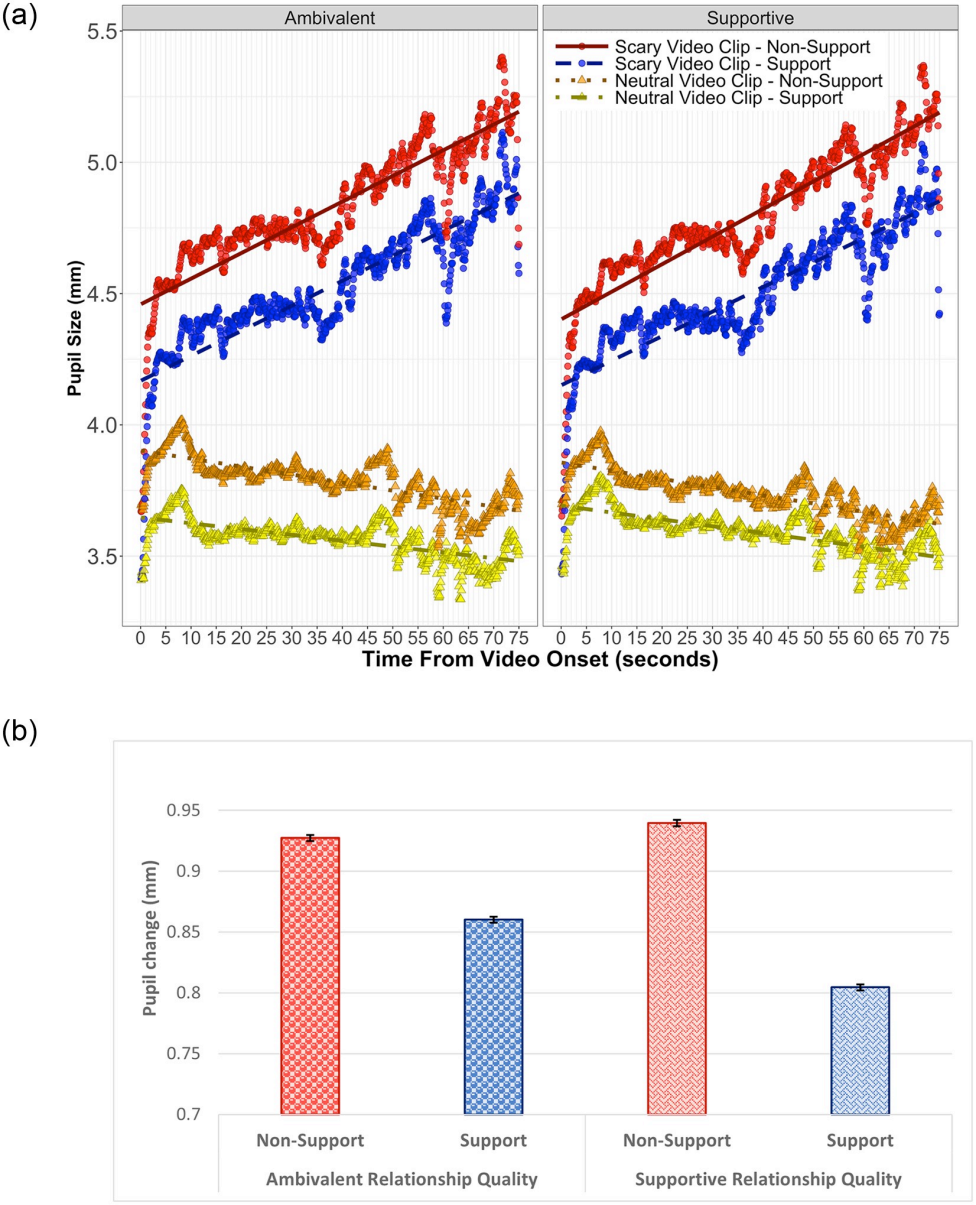
a: *Tonic pupil size as a function of video condition*, *spouse condition*, *and relationship quality*. *Note*. Graph shows pupil size in millimeters across all video clips from videos’ onset to videos’ end. The lines depict the average linear change for all participants and for all video clips as reported in Table 4. Circles and triangles represent the raw data in 100ms bins. b: *Average tonic pupil change by condition*: *Three-way interaction*. *Note*. This graph depicts the amount of increase in pupil size by condition when viewing the horror videos compared to pupil size during neutral videos. Having a supportive marital relationship while concurrently having one’s spouse hold their hand resulted in less pupil dilation during horror videos. Graph depicts the same data as Fig 2a but modeled as change scores to represent more clearly this statistically significant three-way interaction.

We analyzed tonic pupil size, defined as overall differences in pupil diameter during each of the video clips. Pupil sizes were analyzed as a function of Video Clip (Neutral vs. Horror), Spouse Condition (Non-Support vs. Support), and Relationship Quality (Ambivalent vs. Supportive). These variables were dummy coded, with baseline values being neutral clip, non-support, and ambivalent, respectively. This allowed us to test if tonic pupil size changed as a function of Video Clip and if this change was moderated by the emotional support from the spouse and/or marital relationship quality. Thus, testing our hypotheses required examining the interactions between our variables of interest. For this reason, we elected to use dummy coding for our variables, as dummy coding eliminates the need for multiple planned comparisons to test interactions; the simple effects are tested directly in the model. The final model included nested random intercepts for participant and couple, random intercepts for videos, and random-by-video slopes for Relationship Quality. These results are shown in Table 4 and Fig 3a. Results revealed an effect of Video Condition, indicating that horror video clips elicited a larger pupil diameter than neutral video clips (*b* = 0.88, *SE* = 0.10, *t* = 8.74, *p* < .001). Additionally, we found a significant interaction of Video Condition and Spouse Condition such that holding the hand of one’s spouse reduced the pupil diameter increase for horror compared to neutral video clips (*b* = -0.063, *SE* = 0.0015, *t* = -42.09, *p* < .001). The three-way interaction of Video Condition, Spouse Condition, and Relationship Quality was also significant (*b* = -0.065, *SE* = 0.0023, *t* = -28.81, *p* < .001), indicating that the stress buffering effects of handholding were even greater when the marital relationship was supportive compared to ambivalent (see Fig 3b).

**Table 4:** Tonic pupil analysis.

	*b*	*SE*	*t*	*p*-value
Intercept	3.81	0.12	30.91	< .0001 *
Spouse Condition = Support	-0.19	0.15	-1.30	.20
Video Condition = Horror	0.88	0.10	8.74	< .0001 *
Relation. Quality = Supportive	0.022	0.13	0.16	.87
Interaction:	-0.063	0.0015	-42.09	< .0001 *
Spouse Condition = Support X				
Video Condition = Horror				
Interaction:	-0.0002	0.021	-0.001	.99
Spouse Condition = Support X				
Relation. Quality = Supportive				
Interaction:	-0.0052	0.0075	-0.69	.50
Video Condition = Horror X				
Relation. Quality = Supportive				
Interaction:	-0.065	0.0023	-28.81	< .0001 *
Spouse Condition = Support X				
Video Condition = Horror X				
Relation. Quality = Supportive				

*Note*. Relation. = Relationship. Dummy coded baseline comparison groups were: Video Condition = neutral clip, Spouse Condition = non-support, and Relationship Quality Condition = ambivalent.

* *p* < .05.

#### Phasic analysis

Per this study’s preregistration, we investigated specific time-stamped pupil dilation reactivity. We assessed the acute physiological reaction to the individual frightening jump scenes within each horror video clip and its matched neutral video clip. In order to model the phasic pupil response to the individual frightening jump scene stimuli, growth curve analysis was employed [90]. This analysis fits linear and quadratic functions for Time beginning at 750ms after stimulus onset to the pupil size data to capture the change in pupil size over 2500ms. Prior to 750ms pupil changes represents a reflexive pupillary response to the onset of the visual stimulus [68]. Each of the video clips contained at least one frightening jump scene (some had up to four). Each of these frightening scenes were coded for the start of the scene for each video clip. These same time stamps were used for the matched neutral clips to compare between video stimuli. Statistical interactions between the Time function and Video Condition (Horror, Neutral) reveal differences in the pupillary response to the different types of video stimuli. The final model included nested random intercepts for participant, for couple, and for video. Consistent with the tonic analysis, results showed that pupils were larger in horror videos (*b* = 0.98, *SE* = 0.11, *t* = 8.8, *p* < .001). In addition, participants’ pupils were smaller when their spouse was holding their hand (*b* = -0.21, *SE* = 0.1, *t* = -2.05, *p =* .043). These two variables interacted, indicating that the effect of Video Condition was weaker when the participant was receiving emotional support (*b* = -0.093, *SE* = 0.0038, *t* = -24.6, *p* < .001). The linear effect of Time was significant for neutral videos (*b* = -3.64, *SE* = 0.58, *t* = -6.3, *p* < .001), but a significant interaction with Video Condition indicated that this effect went away for horror videos (*b* = 2.81, *SE* = 0.84, *t* = 3.35, *p =* .0008). While the quadratic effect of Time was not significant for neutral videos (*b* = 0.36, *SE* = 0.58, *t* = 0.63, *p =* .53), a significant interaction with Video Condition indicated that in the horror videos, the ‘frightening jump scare’ elicited a short-term increase in pupil size (*b* = -8.24, *SE* = 0.84, *t* = -9.87, *p* < .001). However, there was no significant three-way interactions between time, Video Condition, and Spouse Condition, indicating that the effect of spousal emotional support was limited to tonic measures of pupil size.

#### Post Hoc STAI analysis

Our secondary analysis of participants’ state anxiety indicated there was a calming effect of having one’s spouse hold their hand during the horror movies experiment. We were curious to see if there were similar influences on pupil dilation from STAI. We conducted a similar analysis to our Tonic analysis (described above) but included the STAI measure in addition to Video Condition and Spouse Condition. Results revealed that there was no significant effect of STAI on pupil dilation for neutral videos in the non-support condition (*b* = .077, *SE* = .065, *t* = 1.19, *p* = .24) nor in the spouse support condition (*b* = -.026, *SE* = .098, *t* = -.26, *p* = .79). However, the STAI moderated the effect of pupil dilation during the horror videos such that, as participants reported higher pre-experiment state anxiety, there was a larger pupil dilation effect during the horror videos (*b* = .063, *SE* = .0007, *t* = 88.85, *p* < .001). Additionally, there was a three-way interaction of STAI, Video Condition, and Spouse Condition (*b* = -.058, *SE* = .001, *t* = -51.31, *p* < .001). Participants who reported more state anxiety had larger pupil dilation during horror videos but only for the non-support condition. Pupil dilation was much smaller for those with higher reported anxiety when their spouse was holding their hand during horror videos.

## Discussion

The purpose of the current experiment was to conceptually replicate our previous study’s findings of the effects of spousal emotional support in dampening the ANS’s acute stress response. This dampening effect was observed via pupillometry from a generalizable, real-life stressor: horror movies. We also aimed to extend these results by including the effects of marital relationship quality using a multidimensional scale. Pupillometry captures precise and immediate changes in the body’s ANS stress response and frames a potentially fruitful method to investigate the causal mechanisms linking supportive marital relationships and health. Results from the current experiment revealed significant differences between the support and non-support conditions as well as marital relationship quality conditions. These differences were seen via tonic pupillary responses and self-report measures. Secondary analysis measures of self-report anxiety also revealed significant differences between the spouse conditions. However, the BP analyses did not reveal differences between the experiment conditions. The stress buffering hypothesis asserts that social relationships are beneficial in reducing stress-evoked reactivity during periods of acute stress. The results from the current experiment are in line with this theory. These results extend the current literature by using an innovative method to investigate these associations and provide additional insights into the speed and precision at which these effects happen and can be captured. To our knowledge, this is the first study to investigate the stress buffering effects of emotional support from real-life stressors and the moderating effects of relationship quality on the pupillary stress response.

### Pupillary responses to horror vs neutral videos

Our first hypothesis was that participants exposed to the experiment stressor (i.e., horror video clips) would demonstrate increased pupil dilation compared to the control stimuli (i.e., neutral video clips). Horror movies were chosen as the experiment stressor in the present study because of their ubiquitous applicability and generalizability; watching horror movies is an activity people regularly participate in. Additionally, they have been reliably shown to elicit a physiological reaction [46, 52, 53]. This hypothesis was supported as shown by both the tonic and phasic pupil analyses. The tonic pupil analysis revealed that participant’s average pupil diameter during the horror video clips was larger compared to the neutral video clips. From the phasic analysis we found that even within specific segments of the horror and neutral video clips, which were matched in both length and phasic onset time, there was significant differences between the two video conditions.

Cognitive load or mentally stimulating activity, such as solving arithmetic problems or reading a passage, can enlarge pupil dilation [91–93]. However, if the observed effects were cognitive load and not fear/stress reactivity, we would not expect to see differences between the horror and neutral videos; both videos contained elements of attention demand and cognitive processing (e.g., video sequences, dialogue, narration, and observational stimuli). This finding indicates that during the horror videos there is more ANS reactivity happening beyond that of just cognitive load. Although interesting, the confirmation of this hypothesis is more of a necessary steppingstone in the process rather than the end goal. We were primarily interested to see if this effect can be moderated by spousal emotional support and marital relationship quality. Neither of these constructs could be investigated without confirmation of this hypothesis first.

### Pupillary responses dampened by spousal emotional support

Our second hypothesis, which is part of the contributing novelty of this experiment, was that participants who received spousal emotional support in the form of handholding during the horror video clips would show a weaker pupillary stress response compared to participants without their spouse’s support. Nearly two thirds of the participants in the support condition reported that their spouse’s handholding was supportive to them during the experiment. Whereas pupillometry has been a validated form of measuring ANS activity [73, 94], fear [55, 56, 70, 95], and stress [69, 96], there has been no work, that the authors are aware of, which investigates the stress buffering hypothesis and pupil dilation.

Consistent with this hypothesis, we found that holding hands with one’s spouse while watching a horror movie resulted in a smaller ANS response as measured by tonic pupil dilation. We found that participants who did not have spousal emotional support had larger average pupil diameter over the course of all video clips than those who did have this support. This finding is congruent with the stress buffering hypothesis that receiving spousal emotional support is beneficial during stressful events. We show that, in accordance with the social support literature, spousal emotional support during exposure to a stressor can dampen physiological reactivity. In addition to other well-established biomarkers of physiological reactivity (e.g., cardiovascular, skin conductance, immune functioning, etc.) we show that pupil dilation is sensitive to the effects of stress buffering. These pupillometry findings additionally reveal the speed at which stress buffering occurs, suggesting that the dampening effects of social support are immediate.

The phasic analysis did not reveal a significant difference between the non-support and support conditions for specific instances of frightening jump scenes within the horror videos. The data shows that in a global and general (tonic) sense, having one’s spouse hold their hand is powerfully protective, from experiment onset to end. This stress buffering effect was already in place and thus no additional buffering was evident when adding in specific time-stamped events. In other words, the smaller tonic pupil size overall meant that frightening jump scenes in the horror video were already dampened by spousal support before they even occurred, so that the peak dilation was smaller when spousal support was provided.

In our previous work, we found phasic support for the pupillary stress buffering effects of spousal support. Although results from this study differ, we do not think they are in contention, rather, both are revealing important and different aspects of this association. In the present experiment, participants were not asked to do anything in response to the experiment, simply to watch the videos as presented, whereas in our prior work, participants had to evaluate, respond to, and were evaluated on their responses to cognitively challenging trials of the Stroop task [97]. There may be something inherently different with having to physically respond compared to simply watching or viewing. Additionally, evaluation apprehension [98] may play a critical role in phasic reactivity.

### Spousal emotional support: Secondary measures

Beyond the objective measures of pupillary dilations, participant’s subjective stress and anxiety, as reported from the STAI measure, supported our hypothesis that having spousal emotional support would buffer participant’s stress response. While there was no difference between the non-support and the support conditions for the STAI pretest, the non-support condition reported significantly higher feelings of state anxiety when responding to the STAI post-experiment, whereas the support condition reported no differences pre- and post-experiment. This shows that participants entered the experiment with similar levels of apprehension but the actual exposure to the horror videos was more intense for those without the support of their spouse.

Post hoc analyses confirmed these findings in relation to pupil dilation as well. Participants who felt more anxious prior to the experiment had increased pupil dilation during the horror videos than those who reported less anxiety. Importantly, and in line with our other findings, this effect was moderated by spousal support; participants reporting more anxiety who had their spouse supporting them, had less pupil dilation during the horror videos than those without their spouse’s support. These findings demonstrate that having a spouse’s support—especially when anxiety is high—can dampen both autonomic and affective reactivity to stressors.

Importantly, so as not to influence participant’s responses in the support condition due to the presence of their spouse (e.g., impression management; trying to show that they were not frightened, etc.), participants responded to all self-report measures separated from their spouse. This was done to prevent any perceptions of judgment. Additionally, participants were not informed that there were two spouse conditions (i.e., spouse support condition and non-support condition), to discourage participants from responding to fulfill researcher expectations. Supporting the objective pupillary data, even at a self-reported and reflective level, having spousal emotional support helped to buffer stress.

We found significant BP differences within the participant roles (i.e., being the experiment participant vs. the support providing participant). When participants in the support condition watched the horror videos, their BP was higher compared to when the same participant was providing support to their spouse. This indicates that the study’s experiment did evoke both cardiovascular reactivity and pupillary reactivity.

Interestingly, this hypothesis was not supported by our secondary analyses, cardiovascular data. We did not find differences between participant’s baseline BP and their BP while watching the horror movies. Nor did we find significant differences between the non-support and the support conditions. Although previous research has reliably shown that social support can lower BP reactivity [99–101], we did not find such an association. The lack of cardiovascular differences between the non-support and support conditions in the present experiment could possibly be addressed by the study’s design. This was an exploratory study aimed at the effects of pupillary changes. Pupillary measures were recorded every 1200Hz for each of the 166 participants, resulting in over 14 trillion data points. This provided high precision for the pupillary analyses. In contrast, cardiovascular measures were a secondary analysis aimed at assessing acute stress reactivity generally, with only a few readings. In addition, the selected passive stressor, rather than active, used in this experiment was deliberate to extend our previous study’s results. Prior research though, has found that active stressors tend to evoke more cardiovascular responses than passive stressors [60, 102]. Thus, the lack of cardiovascular effects between these conditions is somewhat unsurprising given the design and aims of the study.

Additionally, anticipated participant procedures and their task apprehension could have played a significant role in the lack of BP effects. There were differences between the participant roles but not between baseline and mid-experiment, this suggests that participant baseline BP could have already been elevated when measuring their baseline BP. Thus, the baseline and mid-task BP change scores may not have reflected accurate reactivity. As part of the study’s recruitment and informed consent, participants were told they would be viewing a series of videos potentially frightening in nature. Tomaka et al. [86] suggests that cognitive appraisals play a central role in the elicitation of threat and challenge responses to potentially stressful situations. It could be that participants were primed by the study’s procedural details and this elevated their baseline BP.

Other potential explanations could be due to the varying temporal sensitivity of these methods. Ditzen and Heinrichs [9] explain that autonomic activation can be assessed through various physiological markers, such as PR, BP, or skin conductance, but that these measures do not necessarily correlate. Indeed, Leuchs et al. [56] found that pupillometry measures were only weakly correlated (and in some cases did not correlate) with skin conductance and electromyography measures. It could be that different methods capture activation of different neurological sub-systems [103, 104].

### Moderating effects of relationship quality

Our final hypothesis extended the results from previous work by adding in an additional condition to the study protocol. Prior to qualifying for the experiment, participants completed the SRI to assess multidimensional relationship quality. Based on their answers, we then categorized participants into the relationship quality conditions of supportive or ambivalent. We hypothesized that participants in the spousal support condition, who also reported their current marital relationship quality to be supportive, would show less pupillary response than those reporting an ambivalent relationship. Thus, we expected to see that marital relationship quality would further dampen the ANS pupillary response to the experiment stressor.

Results from the tonic analysis revealed that the stress buffering effects of handholding were even greater when the marital relationship was supportive compared to ambivalent (i.e., there was a three-way interaction of Video Condition, Spouse Condition, and Relationship Quality). Not surprisingly, relationship quality had no effect in the non-support condition; we would expect that a spouse would have to be present, or at least made salient, for stress buffering to be influenced by relationship quality from an immediate stressor. Although perceived social support, or the perception that support is available if needed, has been shown in the literature to be beneficial [105], this experiment directly manipulated received support. Participants in the study were unaware of the experimental manipulations and thus had no salience to the relationship quality conditions of the study. This important finding demonstrates the value of not only receiving support from one’s spouse, but that the quality of the marital relationship is an important facet in stress buffering. Additionally, this finding is consistent with the study by Coan et al. [14] in their study using functional magnetic resonance imaging. They found spousal emotional support in the form of handholding was most influential when threatened by electric shock if the couple had a good marital relationship.

An important aspect of the effect of relationship quality found in the present study is that we did not categorize marital relationships on a continuum of simply “good” or “bad” as most marriages contain varying degrees of positivity and upsetting aspects simultaneously. Thus, we were interested to see if this more nuanced approach to marital relationship quality is meaningful in pupillary stress buffering effects. Recent research has examined ambivalence in marriage and found those who report spousal ambivalent behavior have worse psychological and physiological health [29, 36–39]. The present study’s finding supports this literature, and we echo previous researchers in urging future studies to include multidimensional measures of relationship quality [35, 106]. Additionally, our results extend this literature by exploring a unique method by which to assess the speed at which stress buffering occurs: pupillometry.

### Limitations

There are several limitations to this study. This experiment was part of a program of study that is exploratory and, to our knowledge, the first to examine the stress buffering effects of spousal emotional support on pupillary reactivity. Although we conceptually replicated much of our initial findings, the use of pupillometry to assess these constructs is novel. More work, from different labs, is needed to confirm and extend our findings. Additionally, the present experiment was limited in data collection due to Covid-19. Although adequate to detect significant differences, it would have been ideal to collect our target sample size.

As noted, pupillometry is an efficient method for physiologically detecting stress and is comparable with traditional methods (e.g., skin conductance, heart rate variability) [69]. Pupillometry provides new empirical data beyond these traditional methods in its near instantaneous measurement capabilities. However, along with these other traditional measures, pupillometry is still a peripheral rather than a pure measure of ANS activity. Methods such as adrenal medullary catecholamines and cortisol measurement [107, 108] would have an interpretive advantage but with invasive and immediacy drawbacks. Our results should be interpreted with caution because of their peripheral nature of measurement.

The horror video clips may not have been very believable since slasher films may not carry a high level of realism (i.e., most people do not expect to be stalked by a masked madman with a hook while running through a crowded street). A realistically stressful or frightening event is difficult to replicate in small video segments; however, it is valuable to know that even startling or surprising situations are managed best with a spouse. Even though the horror films themselves were not highly realistic, they still provided a relatively suspenseful feeling especially in the cinematography compared to the neutral film clips. This could have contributed to the increase in pupil dilation for the horror videos compared to the neutral videos.

The segmented nature of the video clips also prevented participants from gaining a more complete picture of the storyline, and beyond the clips themselves, the way the participants viewed the videos was not necessarily realistic. Most people likely watch horror movies in the dark and perhaps on a couch, whereas this study was conducted in a controlled lit room with participants sitting at a desk. Also, because each horror video clip contained at least one frightening jump scare, and the horror videos were clearly differentiated from the neutral video clips, participants could have anticipated the frightening jump scenes. This anticipation could have precipitated the time interval of the jumps scare scenes and reduced participant’s pupillary response. Had participants watched the horror film in its entirety, they could have developed an understanding and familiarity with the characters and their motives. This could contribute to the intensity of the frightening jump scenes. Such factors could have reduced participant’s responsiveness to the specific frightening jump scenes. This could have led to the observed null effects in the phasic pupil analysis.

### Future directions

As the support condition simultaneously manipulated spousal presence and handholding, it is possible that the observed effects were elicited simply by the physical touch of handholding or only by the presence of their spouse, or possibly both are necessary contributing factors. Future studies could examine these factors independently. This experiment’s aims were to examine provision of support only, however, the perception that one has support if needed has been shown to be an important aspect in stress buffering too [12, 105]. Additionally, future studies could investigate different sources of social support. For instance, testing whether other support members such as other family, close friends, acquaintances, or even strangers elicit results similar to those found in the current study. Indeed, Kamarck, Manuck and Jennings [109] has shown that social support from a friend’s presence during active stressors reduces cardiovascular reactivity.

Results from our previous study [62] and the present work are, to our knowledge, the first studies to investigate these associations. Due to this, the practical application of these findings should be interpreted with some degree of caution. With that in mind, future research could address practical applications in clinical settings. Phasic pupil analyses are optimal for specific, instant, and time-dependent measuring of acute stress (and from our results—stress-buffering). This provides the opportunity to isolate target stimuli and their independent effects. To illustrate, as part of a couple’s therapy session, the influence of different stressors, communications, reactions, or support provision/reception may be variably impactful. Because pupil dilations are instantaneous and recovery time between each stimuli is merely seconds, each of these responses could be segmented and clinicians could analyze each of the independent effects. This could allow the therapist/clinician to identify individual problem areas within the relationship and create a personalized plan for the couple based on their reactions to various stimuli. This facilitates clinician objectivity and acute precision to compliment and augment their clinical acumen and intuition.

## Conclusion

The present preregistered experiment is a conceptual replication and extension. We found that horror movie clips elicit a larger ANS response as seen via pupil dilation than neutral movie clips. Additionally, we found that having spousal emotional support in the form of handholding dampens this pupil dilation and that having a supportive marital relationship reduces this effect even more. These findings contribute to the literature by observed effects with a multidimensional relationship quality scale and by demonstrating the near instantaneous speed at which stress buffering occurs. These results are directly applicable to married couples: if one wants to experience less stress reactivity while watching a horror movie, watch it while holding their spouse’s hand. In addition, they should take a moment before the movie begins to ensure that they have a supportive marital relationship.

## Supporting information

S1 Fig Procedural diagram. *Note*. Eligible couples were randomized to either the support or non-support condition. In the Support condition the participant was positioned in front of the eye tracker and their support giving spouse was positioned across from them behind the eye tracker, so the supporting spouse was unable to see the screen. In the Non-support condition, spouses came in separately from each other (within 72 hours) and completed the tasks alone.
(DOCX)

S1 File Timestamps. (DOCX)
