# Supplementary material for: Spousal emotional support and relationship quality buffers pupillary response to horror movies
Source: PLoS One. 2021 Sep 15;16(9):e0256823. doi: 10.1371/journal.pone.0256823 (PMC8443030; doi:10.1371/journal.pone.0256823)
Supplement: S1 Fig — Note. Eligible couples were randomized to either the support or non-support condition. In the Support condition the participant was positioned in front of the eye tracker and their support giving spouse was positioned across from them behind the eye tracker, so the supporting spouse was unable to see the screen. In the Non-support condition, spouses came in separately from each other (within 72 hours) and completed the tasks alone. (DOCX) [file pone.0256823.s001.docx]

S1 Fig. *Procedural Diagram*


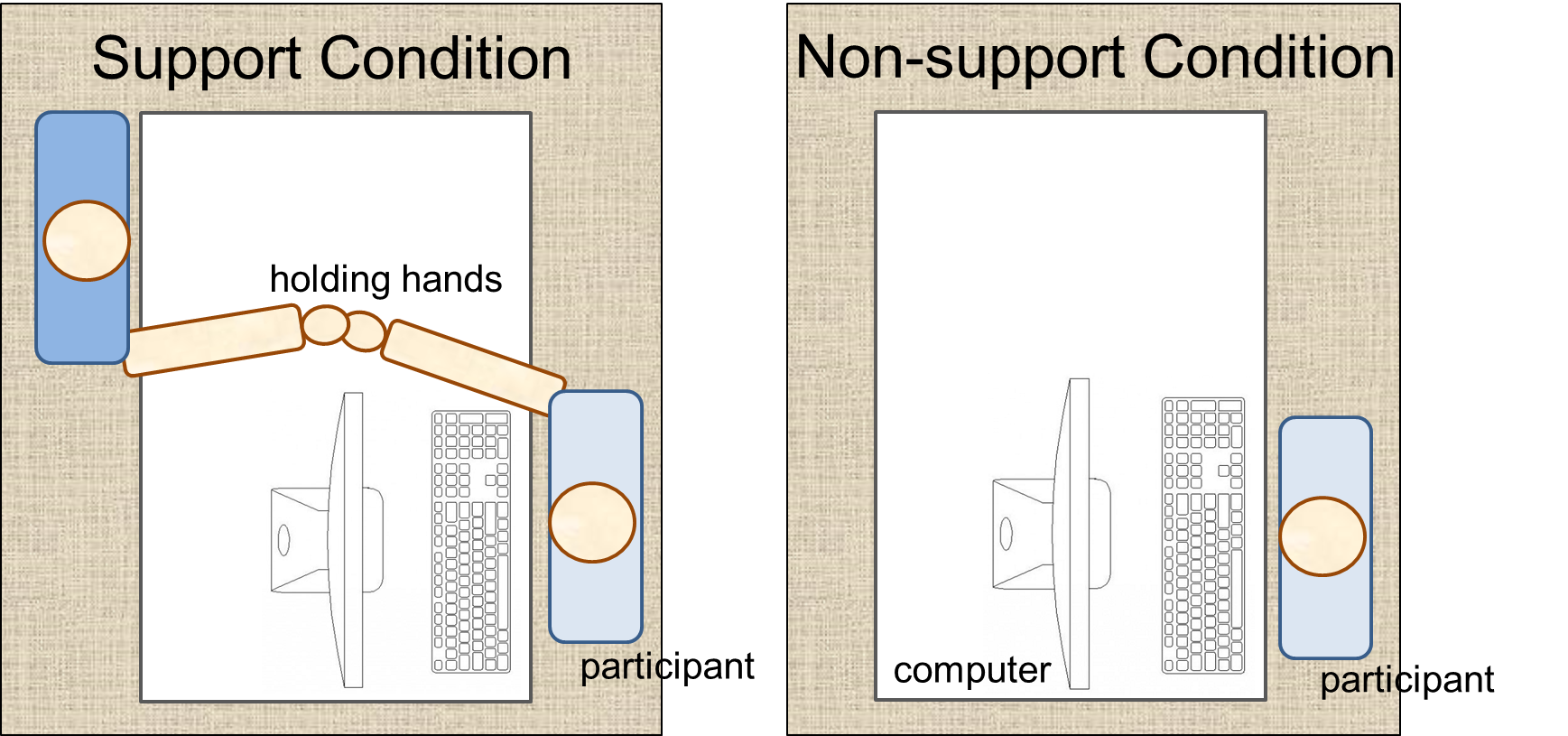


*Note.* Eligible couples were randomized to either the support or non-support condition. In the Support condition the participant was positioned in front of the eye tracker and their support giving spouse was positioned across from them behind the eye tracker, so the supporting spouse was unable to see the screen. In the Non-support condition, spouses came in separately from each other (within 72 hours) and completed the tasks alone.
