# Supplementary material for: Spousal emotional support and relationship quality buffers pupillary response to horror movies
Source: PLoS One. 2021 Sep 15;16(9):e0256823. doi: 10.1371/journal.pone.0256823 (PMC8443030; doi:10.1371/journal.pone.0256823)
Supplement: S1 File — (DOCX) [file pone.0256823.s002.docx]

S1 File. *Timestamps*

Gillespie, J. (1997). I know what you did last summer. Columbia Pictures, Culver City, CA.

- Clip 1: 1:11:55-1:13:08 Police Office Murdered
- Clip 2: 1:16:34-1:17:05 Cashier Murdered
- Clip 3: 1:17:10-1:18:20 Murderer Disguised as Mannequin
- Clip 4: 1:20:57-1:21:53 Murder in Alley
- Clip 5: 1:49:05-1:49:47 Attacked in Shower

Cannon, D. (1998). I still know what you did last summer. Columbia Pictures, Culver City, CA.

- Clip 1: 1:15:41-1:15:59 Murder in Kitchen
- Clip 2: 1:17:18-1:17:55 Falling Through Glass
- Clip 3: 1:17:57-1:19:16 Trapped in Greenhouse
- Clip 4: 1:22:12-1:22:51 Bodies in Cellar
- Clip 5: 1:25:14-1:26:10 Stabbing Through Bellhop
- Clip 6: 1:38:57-1:39:56 Pulled Under Bed

Hardesty, T. (1997). Alaska’s wild Denali. Alaska Video Postcards, Inc. Anchorage, AK.

- Clip 1: 0:10:14-0:11:16 Road to Denali
- Clip 2: 0:11:20-0:12:38 Bus Ride to Denali
- Clip 3: 0:13:08-0:14:26 Tourists at Denali
- Clip 4: 0:15:03-0:15:20 Moose Playing
- Clip 5: 0:16:01-0:16:45 Goats on the Mountainside
- Clip 6: 0:21:50-0:22:20 Swans Swimming
- Clip 7: 0:52:33-0:53:10 Bears Eating Grass
- Clip 8: 0:53:20-0:53:57 Bears Digging for Roots
- Clip 9: 0:54:01-0:54:56 Bears and Berries
- Clip 10: 0:55:05-0:56:00 Bear and Caribou
- Clip 11: 0:56:05-0:57:09 Bear Family Wandering
